# Development of an Accurate Double Isotopic Standard LC–MS/MS Method for Hyaluronic Acid Quantification in Biological Matrices

**DOI:** 10.1021/acs.analchem.5c06285

**Published:** 2026-02-11

**Authors:** Simone Manzi, Alessandra Altomare, Giacomo Mosconi, Maria Serena Rossitto, Luciano Messina, Anna Gallo, Marina Carini, Giancarlo Aldini, Giovanna Baron

**Affiliations:** † Department of Pharmaceutical Sciences (DISFARM), 9304 Università degli Studi di Milano, Via Mangiagalli 25, Milan 20133, Italy; ‡ Fidia Farmaceutici S.p.A., Contrada Pizzuta, Noto, SR 96017, Italy; § 18755 Fidia Farmaceutici S.p.A., Via Ponte della Fabbrica 3/A, Abano Terme, PD 35031, Italy

## Abstract

Hyaluronic acid (HA) plays key roles in tissue hydration, repair, and cellular signaling. Its quantification in biological matrices is crucial but challenging due to its endogenous nature and poor mass spectrometric detectability. We developed a robust method based on enzymatic hydrolysis, dual ^13^C-labeled internal standards, and the standard addition method combined with LC–MS/MS analysis. Samples from bovine vitreous humor and human synovial fluid were depolymerized with recombinant hyaluronidase to generate Δ4-mer oligomers, quantified using 100%- and 50%-^1^ ^3^C-labeled HA as internal standards to correct the variabilities of the enzymatic digestion and MS detector. The standard addition method was used to control the matrix effects. The method showed excellent linearity (*r* ^2^ > 0.99), low estimated LOD (0.147 ± 0.007 μg/mL for bovine vitreous humor and 0.143 ± 0.028 μg/mL for human synovial fluid) and LOQ (0.491 ± 0.022 μg/mL for bovine vitreous humor and 0.475 ± 0.093 μg/mL for human synovial fluid) values, high recovery (>90%), and suitable accuracy. Significant matrix effects were detected, reinforcing the need for the standard addition method. HA concentrations measured were consistent with physiological ranges. This validated strategy offers a reliable tool for HA quantification in complex biological samples, supporting both clinical and pharmaceutical applications.

## Graphical abstract

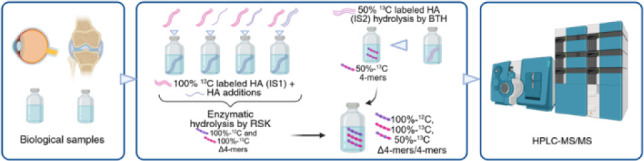

## Introduction

Hyaluronic acid (HA) is a naturally occurring linear polysaccharide characterized by polydisperse repeating disaccharide units composed of alternating d-glucuronic acid and *N*-acetylglucosamine residues connected through β-1,4 and β-1,3 glycosidic linkages.^1^ HA is an integral component of the extracellular matrix, abundantly present in connective tissues, skin, and synovial fluid and fulfilling numerous physiological functions. Owing to its exceptional water-binding capacity, HA significantly enhances tissue hydration and provides structural support and effective joint lubrication. HA plays a key role in organizing the extracellular matrix and modulates fundamental cellular processes, including migration, proliferation, and differentiation, through interactions with specific cellular receptors, such as CD44 and RHAMM. HA is also involved in tissue repair and wound healing processes. Immune responses to HA are strictly related to its molecular weight distribution, displaying either proinflammatory or anti-inflammatory effects. High-molecular-weight HA displays anti-inflammatory and immunosuppressive properties, whereas low-molecular-weight HA exerts potent proinflammatory effects.^2^ Additionally, HA participates in neural development and has been implicated in cancer progression, underscoring its multifaceted role in physiological homeostasis. Platforms based on HA have been developed because it is ideally suited for chemical modifications, usually targeting the carboxyl, hydroxyl, and *N*-acetyl groups. For example, linear HA esterification improves mucosal adhesion, resistance to degradation, and continuous release; cross-linking between chains improves viscoelastic and mechanical properties. Alterations in HA metabolism—such as changes in synthesis rates, fragmentation patterns, and receptor interactions—have been implicated in several pathological conditions. In chronic inflammatory diseases like osteoarthritis and inflammatory bowel disorders, low-molecular-weight HA fragments enhance inflammatory reactions by activating immune cells through receptors such as CD44 and Toll-like receptors. Moreover, elevated HA levels within tumor microenvironments contribute to cancer progression by promoting tumor cell proliferation, invasion, angiogenesis, and metastasis. Abnormalities in HA metabolism also play a role in fibrotic diseases by disrupting normal extracellular matrix organization and impairing the tissue regeneration processes. Similarly, decreased synthesis or accelerated degradation of HA is linked to tissue aging and degenerative joint diseases. HA dysregulation has also been reported in neurological disorders, affecting neuroinflammation and neuronal function. Collectively, these findings establish HA as an important biomarker and a promising therapeutic target across various disease contexts.^3–6^ HA has become an essential component of pharmaceutical therapies largely owing to its high biocompatibility, biodegradability, and diverse therapeutic characteristics. Clinically, HA is frequently administered through intraarticular injections for osteoarthritis management, effectively relieving joint discomfort and enhancing function. Additionally, it finds extensive use in ophthalmic applications, particularly for treating dry eye conditions and facilitating surgical procedures involving ocular tissues. In dermatology, HA promotes wound healing and tissue regeneration and is extensively utilized in cosmetic interventions aimed at improving skin hydration and elasticity. Its capability to influence inflammatory responses and serve as an effective drug-delivery medium further highlights its therapeutic versatility and potential. Quantifying HA in biological samples is essential for research and clinical applications due to its involvement in numerous physiological processes and its relevance in various diseases. Accurate quantification and characterization of HA, including its molecular weight distribution and concentration, provide essential insights into tissue health, inflammatory processes, and disease progression. HA analysis facilitates diagnosis, prognosis, and therapeutic monitoring in diseases such as osteoarthritis, cancer, and chronic inflammatory conditions. Moreover, detailed HA profiling supports the development and quality control of pharmaceutical formulations and biomaterials. Thus, robust analytical methods are indispensable to advancing our understanding of HA’s biological functions and therapeutic potential. Numerous analytical methods have been employed to measure HA concentration in biological matrices, each with distinct advantages and limitations.^7,8^ The analysis of HA is generally challenging due to the chemical nature of the analyte: it is a high-molecular-weight, nonvolatile polymer lacking chromophore groups and exhibiting negligible ionization efficiency in mass spectrometry. As a result, HA is not easily detectable in its native form without specific depolymerization and derivatization. Currently, the state-of-the-art method for measuring HA in biological matrices is based on liquid chromatography coupled with electrospray ionization mass spectrometry (LC–ESI-MS)^9^ or capillary electrophoresis coupled to MS,^10^ combined with extensive sample preparation. This typically includes HA purification, enzymatic hydrolysis, derivatization of the resulting oligomers, and analysis by LC–ESI-MS or CE–MS in multiple reaction monitoring (MRM) mode. Two major drawbacks can be identified in the current methods. The first, as also pointed out by Volpi et al.,^9^ is the variability in the efficiency of enzymatic digestion—a critical aspect of the procedure; any variation in this step directly impacts the yield of the oligomers, which are subsequently analyzed following the derivatization protocol. Several factors can influence the enzymatic activity, including the quality of the enzyme, its stability during storage, and its intrinsic activity within the sample, which may vary due to sample heterogeneity. To account for such variations, we propose including isotopically labeled HA, uniformly labeled with ^13^C (i.e., all ^12^C atoms replaced by ^13^C), to the sample prior to enzyme addition. Variations in enzymatic activity would affect both the native analyte and its isotopic analogue equally, thereby enabling the normalization of these fluctuations. The second drawback of the current methods is the absence of an isotopic analogue of the analyte to serve as an internal standard. In MS-based quantitative analysis, the use of an internal standard is essential due to the inherent instability of the mass spectrometry response. In the present paper, an isotopic analogue of the analyte with 50% of C labeled with ^13^C is added to the sample before analysis.

## Materials and Methods

### Chemicals

Formic acid, ammonium formate, acetonitrile (ACN), methanol, LC–MS grade solvents, and bovine testicular hyaluronidase (BTH) were purchased from Sigma-Aldrich (Merck Life Science S.r.l., Milan, Italy). HPLC-grade water (18 MΩ cm) was purified with a Milli-Q system (Millipore, Bedford, MA, USA). 700 kDa hyaluronic acid and recombinant bacterial hyaluronidase from *Streptomyces koganeiensis*, rHyal-SK (RSK) were supplied by Fidia Farmaceutici S.p.A., Abano Terme, Padova, Italy. d-glucose (U–^13^C_6_, 99%) was obtained from Cambridge Isotope Laboratories Inc. (Andover, MA, USA). Bovine vitreous humor was from the company Innovative Research (Peary Court, Novi, MI 48377, United States). Human synovial fluid from a healthy single male donor was from CliniSciences (Guidonia Montecelio – Italy).

### Synthesis of HA Isotopic Standards

Labeled HAs were biosynthetically prepared using *Streptococcus equi* subsp. *equi* with d-glucose (U–^13^C_6_, 99%) as the carbon source. The strain used in this study is the property of Fidia Farmaceutici S.p.A. (Noto Unit collection) and belongs to the genus *Streptococcus* routinely used for HA production, species *equi* subsp. *equi*.^11–13^ The detailed experimental procedure is provided in the Supporting Information (S1).

### Characterization of HA Isotopic Standards

The isotopic labeling of the two HA standards was verified by HRMS analysis of the isotopic patterns of the tetrameric species obtained by enzymatic hydrolysis catalyzed by RSK, as described in the Supporting Information (S2). Oligomers obtained by enzymatic hydrolysis were then analyzed by LC–ESI-HRMS. Chromatographic separation was carried out using a Hypersil GOLD HILIC column (particle size 3 μm, internal diameter 2.1 mm, length 150 mm, Thermo Scientific), protected by a Hypersil GOLD HILIC precolumn and maintained at 40 °C with an LC-30 UHPLC system (AB Sciex). An aliquot of 10 μL was injected and eluted with a multistep gradient of mobile phase A (100 mM ammonium formate, pH 3) and mobile phase B (ACN), as described in the section HPLC–MS Conditions. HPLC was connected to a hybrid quadrupole-time-of-flight (QTOF) mass spectrometer (X500R QTOF, AB Sciex, Milan, Italy) operated in information-dependent acquisition (IDA) mode to acquire both full MS and MS/MS spectra. The instrument, equipped with an ESI source (Turbo V), performed negative ion mode analysis with the following source parameters: spray voltage: −4.5 kV, ion source gas 1: 40 psi, ion source gas 2: 55 psi, gas temperature 2: 550 °C, curtain gas: 30 psi, CAD gas: 7, declustering potential (DP): −80 V, DP spread: 0 V, collision energy (CE): −10 psi, CE spread: 0 V. The instrument was set to acquire full MS spectra with the QTOF analyzer at a resolution of 30,000 (fwhm at *m*/*z* 400), scanning in the 200–1800 *m*/*z* range and with a buildup time of 0.25 s. For the fragmentation of each full scan ion, a collision energy of −35 V (CID) with a CE spread of 15 V was used, and the MS/MS spectra acquired by the TOF analyzer were set to fragment the ten most intense ions (intensity >500 cps) of the previous full MS event according to the following parameters: profile mode (resolution 15,000 fwhm at 200 *m*/*z*), accumulation time: 0.075 s, charge state: 1 to 5, dynamic exclusion activated to exclude ions already fragmented: 2 times in a time interval of 5 s.

### HA Hydrolysis Optimization

20 mg of HA 700 kDa was solubilized in 1000 μL acetate buffer (100 mM, 150 mM NaCl, pH 5.2) and kept for 1 h under mild stirring in a thermomixer (350–400 rpm) at room temperature. Subsequently, an aliquot of 20 μL of RSK from a stock solution (100 U/mg, 100.696 U/mL) was added, and the sample was incubated at 37 °C in a thermomixer under mild stirring (350–400 rpm) for 48 h. An aliquot of the incubation mixture was collected at different time points (0, 0.5, 1, 2, 4, 24, and 48 h), and 10 volumes of cold methanol were added to stop the hydrolysis and promote protein precipitation. Samples were kept for 30 min at −20 °C, then centrifuged at 4 °C for 15 min at 15,200 rpm, and the supernatant was dried under vacuum. The dry sample was reconstituted in 70 μL of 100 mM ammonium formate (pH 3/ACN 50:50% v/v), vortexed, centrifuged for 15 min at room temperature, and the supernatant was transferred to a vial for analysis by LC–HRMS.

### Sample Preparation

For the determination of hyaluronic acid in bovine vitreous humor (BVH) and human synovial fluid (HSF), an initial appropriate dilution in water was carried out because of the high HA concentration in the samples: BVH was diluted 12-fold and HSF was diluted 1:10. An aliquot of 20 μL of the so-obtained samples was mixed with 40 μL IS1 (50 μg/mL), 100 μL of acetate buffer (200 mM, 300 mM NaCl, pH 5.2), and increasing concentrations of HA. Specifically, for BVH, the additions were 0, 4, 12, and 20 μg/mL, whereas for HSF, they were 0, 8, 24, and 40 μg/mL. An aliquot of 2 μL of RSK 2000 U/mL was added to all the samples, and the hydrolysis was then carried out in a thermomixer at 37 °C at 500 rpm for 24 h. The reaction was stopped by adding 10 volumes of cold methanol; samples were kept at −20 °C for 30 min and then centrifuged at 4 °C for 15 min at 15,200 rpm. The supernatant was collected and dried under vacuum at 50 °C. Finally, the dry sample was dissolved in 60 μL of 100 mM ammonium formate (pH 3/ACN 50:50% v/v) to which were added 10 μL of IS2 previously hydrolyzed. The sample was vortexed, centrifuged for 15 min at 15,200 rpm at room temperature, and analyzed by LC–MS/MS as described in the section HPLC–MS Conditions.

### HPLC–MS Conditions

Chromatographic separation was carried out using an Exion LC-100 HPLC system (ABSciex, Milan, Italy) operating at a constant flow rate of 300 μL/min. An aliquot of 10 μL was injected and eluted with a multistep gradient of mobile phase A (ammonium formate 100 mM, pH 3) and mobile phase B (ACN) as follows: 0–0.20 min isocratic 80% of B, 0.20–6.70 min from 80% of B to 30% of B, 6.70–14.70 min isocratic 30% of B, and 14.70–23.00 min isocratic 80% of B. Detection of Δ4-mer (analyte) and isotopic analogues (IS) was carried out by using a Multiple Reaction Monitoring (MRM) method and a triple quadrupole analyzer (API 4000, ABSciex, Milan, Italy) equipped with an ESI source set in negative ion mode and the following parameters: Charged Aerosol Detector (CAD) gas: 4 au, curtain gas: 25 au, gas 1: 40 au, gas 2: 60 au, ion spray voltage: −4500 V, temperature: 550 °C, entrance potential: −10 V. The MRM transitions for the analyte and IS are reported in Table 1 together with instrument parameters optimized following direct infusion of the hydrolyzed standards. The analysis of the data and control of the instrument were performed by means of Analyst and Sciex OS software (ABSciex, Milan, Italy).

**Table 1:** MRM Parameters for the Analysis of Δ4-Mer (Analyte) and Isotopic Analogues (IS)

Compound	Parent ion	Product ion	^a^CE	^a^DP	^a^CXP
Δ4-mer	757	554	–48	–100	–15
Δ4-mer 100% ^13^C (IS1)	785	392	–48	–100	–15
4-mer 50% ^13^C (IS2)	789	199	–48	–100	–15

a CE = collision energy, DP = declustering potential, CXP = collision exit potential.

### Quantitative Analysis

The endogenous content of HA was determined by the standard addition method (SAM); three different concentrations of the target compound and a fixed concentration of IS1 and IS2 were added to equal portions of the same matrix for the construction of a calibration curve. One portion of the matrix was analyzed without any addition of the target compound but still contained the fixed concentrations of IS1 and IS2 in order to account for the signal from the preexisting endogenous compound under the same analytical conditions. The SAM regression equation is expressed as *y* = *ax* + *b*, where “*a*” is the slope of the SAM calibration curve, and “*b*” is the intercept on the *y*-axis, reflecting the response (e.g., peak area ratio) due to the endogenous compound already present in the matrix. When *y* equals zero, *x* = −*b*/*a*, which indicates the negative concentration corresponding to the amount of the preexisting compound in the tested matrix. Calibration curves were built by plotting on the *x*-axis the spiked HA concentrations as μg/mL and on the *y*-axis the following area ratio: *y* = [area­(Δ4-mer)/area­(Δ4-mer 100% ^13^C)]/area­(4-mer 50% ^13^C).

### Method Validation

The method was validated in terms of linearity, LOQ and LOD, matrix effect, recovery, precision, and accuracy intraday and interday, according to Hasegawa et al.^14^ The limits of detection (LOD) and quantification (LOQ) were estimated based on the signal-to-noise (S/N) ratio. Specifically, the height of the analyte peak was measured and compared with the amplitude of the baseline noise immediately before and after the peak. The S/N ratio was calculated by dividing the peak height by the baseline noise height. Given that the concentration of the endogenous compound was previously determined using the standard addition calibration curve, the LOD and LOQ were estimated by scaling the known concentration according to the S/N ratios of 3 and 10, respectively. The LOD was calculated using the following formula:

$$\mathrm{LOD}=C_{\mathrm{measured}}\times \frac{3}{S/N}$$

and the LOQ was calculated as

$$\mathrm{LOQ}=C_{\mathrm{measured}}\times \frac{10}{S/N}$$

where *C*_measured_ is the concentration of the analyte determined by the standard addition method, and S/N is the signal-to-noise ratio observed.

The accuracy and precision (intraday and interday) of the method were evaluated using the standard addition approach due to the endogenous nature of the target analyte. Equal aliquots of each biological matrix were divided into four groups and spiked with increasing amounts of the Δ4-mer (zero, low, medium, and high concentrations) along with fixed concentrations of the internal standards, IS1 and IS2. All samples underwent enzymatic hydrolysis, extraction, and instrumental analysis under the same conditions. The preexisting concentration of the endogenous Δ4-mer (denoted as A) was determined from the unspiked matrix. For each spiked level, the net concentration of the analyte was calculated by subtracting A from the total measured concentration (*B*) in the spiked sample. The accuracy at each concentration level was expressed as the percentage ratio between the net concentration and the nominal spiked concentration according to the formula:

$$\mathrm{Accuracy}\;\left(\%\right)=\left(\frac{B-A}{\mathrm{Nominal}\;\mathrm{spiked}\;\mathrm{concentration}}\right)\times 100$$

Precision was evaluated as the relative standard deviation (%RSD) of at least three replicate measurements at each concentration level, based on the net concentrations (*B* – *A*). Acceptance criteria for both accuracy and precision followed standard bioanalytical validation guidelines, with values within ±15% considered acceptable for all concentration levels, except at the lower limit of quantification (LLOQ), where values within ±20% were tolerated.

The matrix effects and extraction recovery of Δ4-mer in the two biological matrices were evaluated using a comparative approach based on LC–MS/MS peak area measurements, as already reported.^14^ Three independent aliquots of each biological matrix were prepared. The first aliquot was processed as described above, including hydrolysis, extraction, and evaporation, without any analyte addition and was used to determine the endogenous HA concentration (**sample A**). The second aliquot was spiked with HA at a concentration equal to the endogenous level, as determined in sample A and subsequently subjected to the complete sample preparation procedure (**sample B**, pre-extraction spike). The third aliquot was processed without analyte addition through hydrolysis, extraction, and evaporation; after evaporation, the dried extract was reconstituted with 100 mM ammonium formate (pH 3)/ACN (50:50, v/v) containing Δ4-mer derived from the hydrolysis of HA at the same concentration as that spiked in sample B (**sample C**, postextraction spike). All samples were spiked with IS1 and IS2, and 10 μL aliquots were injected into the system.

In parallel, a standard solution of Δ4-mer derived from an amount of HA equal to that contained in sample A, prepared in 100 mM ammonium formate (pH 3)/ACN (50:50, v/v) without matrix or extraction, was analyzed (**sample D**).

Matrix effects were calculated using the formula:

$$\mathrm{Matrix}\;\mathrm{effect}\;\left(\%\right)=\left(\frac{C-A}{D}\right)\times 100$$

Recovery rates were calculated using the formula:

$$\mathrm{Recovery}\;\mathrm{rate}\;\left(\%\right)=\left(\frac{B-A}{C-A}\right)\times 100$$

This approach allowed for the independent estimation of ion suppression and enhancement effects caused by the matrix and the efficiency of the extraction process, even in the presence of endogenous levels of the target analytes.

The matrix added with the internal standard was analyzed after two cycles of freeze–thaw (at −80 °C) to evaluate the analyte stability.

To evaluate method robustness, an aliquot of HA (100 μg/mL) was digested in the absence or presence of IS1 using decreasing concentrations of RSK: 5000, 2000, and 1000 U/mL. For samples digested in the presence of IS1, both the Δ4-mer peak areas and the Δ4-mer/100%-^13^C-Δ4-mer peak area ratios were measured. For samples digested in the absence of IS1, only the Δ4-mer peak areas were measured. Measurement precision was assessed by calculating the Δ4-mer peak area, either unnormalized or normalized to the 100%-^13^C-Δ4-mer peak area (for IS1-containing samples).

### Statistical Analysis

The statistical analysis was carried out using GraphPad Prism 10.6.1 software (GraphPad Software, Boston, Massachusetts, USA).

## Results and Discussion

### Method Overview

Figure 1 illustrates the methodological overview we propose for accurate quantification of hyaluronic acid (HA) in biological matrices, based on the use of a dual internal standard approach. The method has been applied to bovine vitreous humor and human synovial fluid but is adaptable to other biological matrices after appropriate sample preparation steps. Initially, the sample is diluted in water to account for the high analyte concentration typical of the tested biological matrices. Subsequently, the first internal standard, 100%-^13^C-labeled HA (^13^C-HA, IS1), is added, followed by the recombinant bacterial hyaluronidase (RSK). This enzyme catalyzes the depolymerization of both endogenous HA and spiked ^13^C-HA into tetramers (Δ4-mers) and ^13^C-labeled tetramers (100%-^13^C-Δ4-mers), respectively. The inclusion of ^13^C-HA serves to correct for potential variations in enzymatic activity, which may be influenced by factors such as enzyme quality, stability during storage, and differential enzymatic efficiency across heterogeneous samples. A Δ4-mer isotopic analogue containing 14 carbon atoms labeled with ^13^C out of a total of 28 carbon atoms (50%-^13^C-4-mer) is used as a second internal standard (IS2) to normalize the mass spectrometric (MS) response. IS2 was generated by hydrolyzing an isotopic mixture of ^13^C-labeled hyaluronic acid (HA) using bovine testicular hyaluronidase (BTH). The responses of the analyte Δ4-mer and of the two isotopes 100%-^13^C-Δ4-mer and 50%-^13^C-4-mer are then detected by HPLC–MS/MS in MRM mode. Quantitative analysis was performed by the standard addition method.

**Figure 1:**
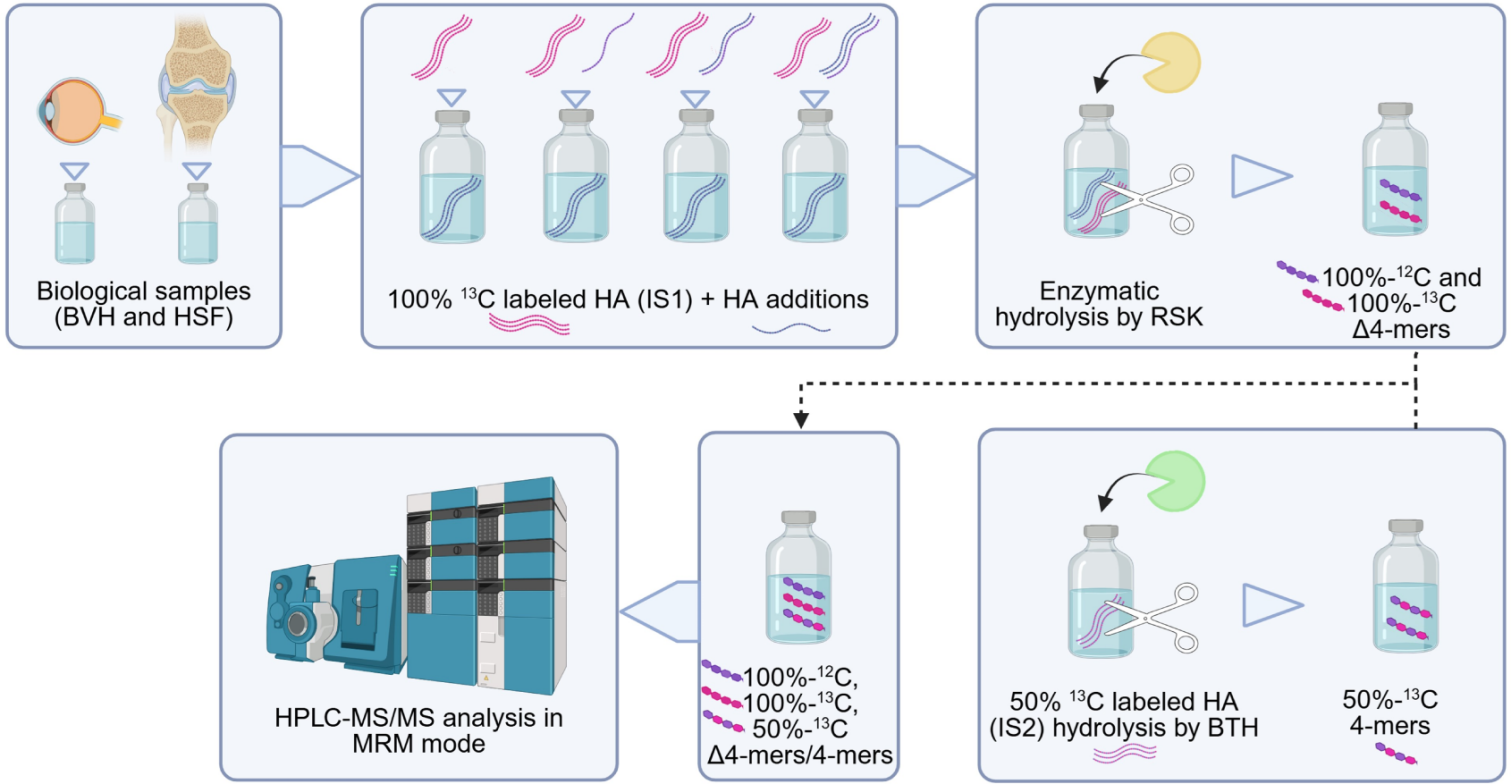
Methodological overview: dilution of biological sample, addition of IS1 and increasing concentration of HA, enzymatic hydrolysis by RSK for 24 h, addition of IS2 previously hydrolyzed by BTH, and HPLC–MS/MS analysis (BVH = bovine vitreous humor; HSF = human synovial fluid; RSK= recombinant bacterial hyaluronidase from *Streptomyces koganeiensis*; BTH = bovine testicular hyaluronidase; HA = hyaluronic acid; Δ4-mers = tetramers obtained by RSK; 4-mers = tetramers obtained by BTH).

### HA Isotope Preparation and Characterization

The advancement of the present method compared to those reported in the literature lies in the use of two internal standards, one for controlling the enzymatic digestion efficacy of HA and the second to normalize the detector response. The isotopic HAs had a molecular weight of approximately 200,000–300,000 Da and an intrinsic viscosity (dL/g) between 6.0 and 12.0. Full characterization of the two HA isotopes was carried out by enzymatic depolymerization, and the resulting Δ4-mer metabolites were then analyzed by high-resolution MS and MS/MS analysis.

Figure 2 compares the high-resolution mass spectra of the Δ4-mer (panel A) and the 100%-^13^C-labeled Δ4-mer (panel B) obtained from the enzymatic hydrolysis of HA and 100% labeled ^13^C-HA, respectively. The monoisotopic peaks are observed at *m*/*z* 757.2169 and *m*/*z* 785.3071, corresponding to the unlabeled and labeled tetramers, respectively. Given that the Δ4-mer contains 28 carbon atoms (C_28_H_42_N_2_O_22_), the observed mass shift confirms that the ion at *m*/*z* 785.3071 results from the complete replacement of all 28 carbon atoms by ^13^C (^13^C_28_H_42_N_2_O_22_).

**Figure 2:**
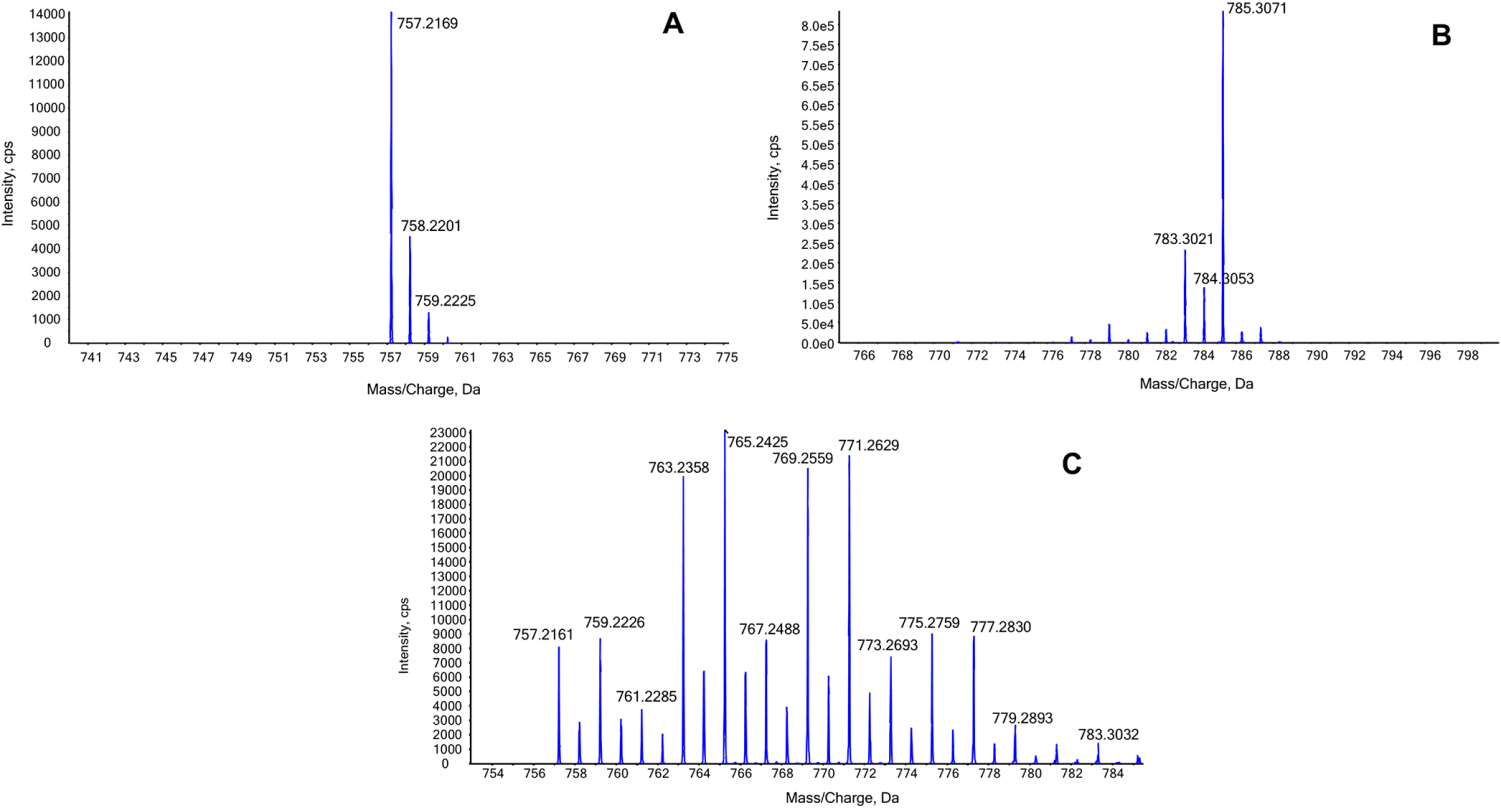
High-resolution mass spectra of: A) Δ4-mer, B) 100%-^13^C-labeled Δ4-mer, and C) 50%-^13^C-labeled Δ4-mer obtained from the RSK enzymatic hydrolysis of HA, 100% labeled ^13^C-HA, and 50% labeled ^13^C-HA, respectively.

The identities of both the unlabeled and labeled Δ4-mer were further confirmed by tandem mass spectrometry (MS/MS) (Figure 3). The MS/MS spectrum of the ion at *m*/*z* 757 displays characteristic fragment ions (157, 175, 396, 554), whose assignments are illustrated in Figure 3 (upper panel), validating the sequence of the four sugar units in the tetramer, namely, D-GA(1)-NAG(2)-GA(3)-NAG(4), where D-GA and GA indicate unsaturated and saturated glucuronic acid, respectively, NAG is *N*-acetyl-glucosamine, and the number in brackets indicates the position of the sugar moiety. In the MS/MS spectrum of the ion at *m*/*z* 785, the observed fragment ions at *m*/*z* 163, 181, 392, 410, and 574 show mass shifts consistent with 100% ^13^C incorporation, further confirming the complete labeling of the molecule.

**Figure 3:**
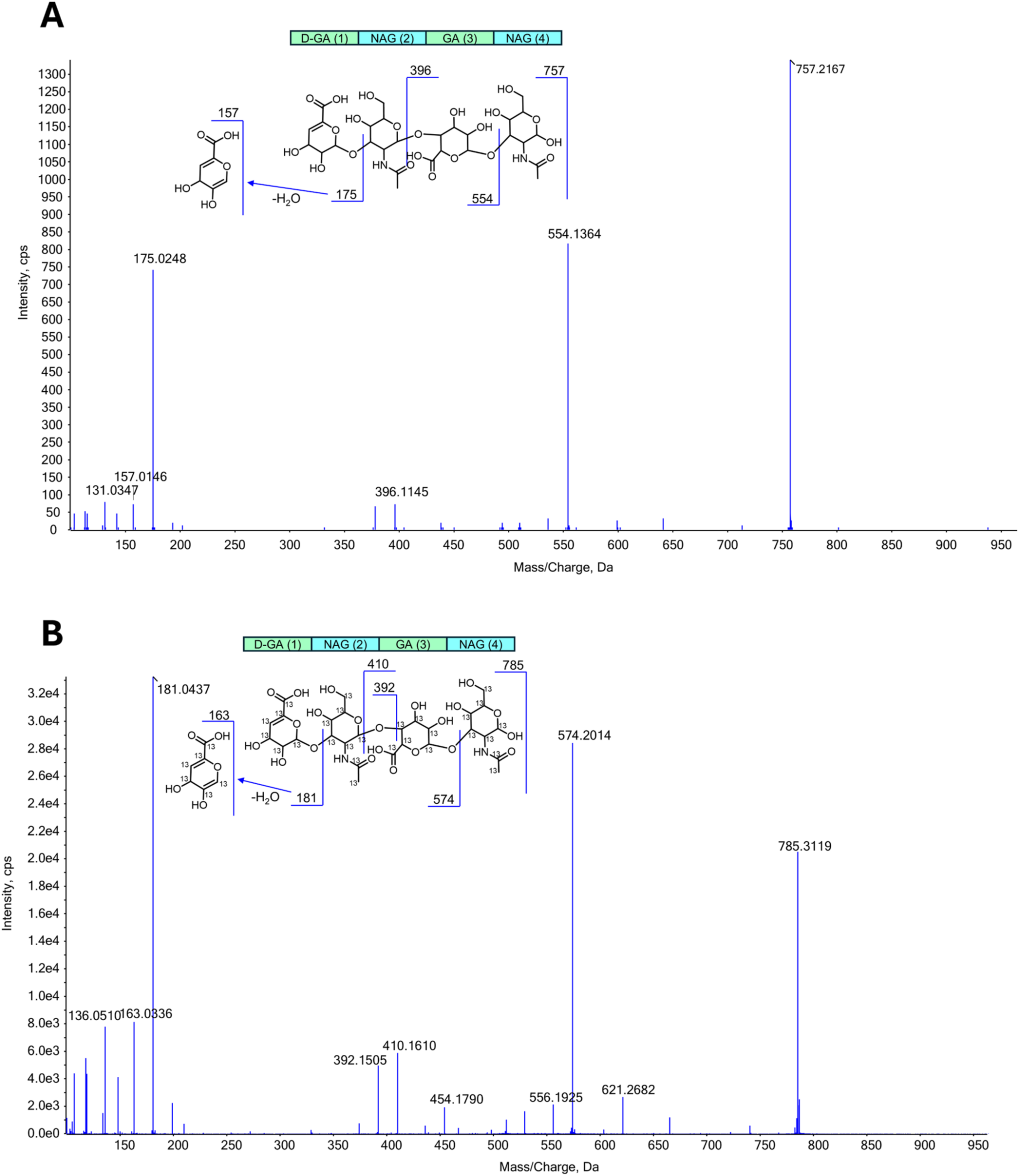
MS/MS spectra of the ion at *m*/*z* 757 A) and *m*/*z* 785 B) refer to Δ4-mer and 100%-^13^C-Δ4-mer.

In addition to the ion at *m*/*z* 785, the MS spectrum of the ^13^C-Δ4-mer also displays two additional ions at *m*/*z* 783.3021 and 784.3053, with relative abundances of 30.1% and 18.9%, respectively. These signals indicate partial ^13^C incorporation, corresponding to 26 and 27 ^13^C atoms incorporated, respectively. According to Rampratap et al.,^15^ such incomplete ^13^C enrichment may arise from variable ^13^C incorporation into the acetyl group of the GlcNAc residue. During the biosynthesis of UDP-*N*-acetylglucosamine from glucose-6-phosphate, an unlabeled acetyl group—originating from unlabeled carbon sources present in the culture medium—may be transferred by the acetyltransferase enzyme. To investigate this hypothesis, we performed MS/MS analyses. The MS/MS spectrum of the ion at *m*/*z* 785 reveals two fragments at *m*/*z* 181 and 163, corresponding to the fully ^13^C-labeled unsaturated glucuronic acid unit (D-GA(1)). These same fragments were also observed in the MS/MS spectra of the parent ions at *m*/*z* 783 and 784, suggesting that partial ^13^C incorporation does not affect the D-GA(1) moiety. The MS/MS spectrum of the ion at *m*/*z* 783 shows a set of peaks which differ by 2 mass units, namely 408 and 410, 390 and 392, 572, and 574. These doublets can be attributed to MS/MS fragments containing the acetyl moiety, which can contain carbon atoms either as ^12^C or ^13^C. Figure 4 shows the MS/MS spectrum and assignments. To definitively confirm that the residual ^12^C atoms are located within the acetyl moiety, an aliquot of ^13^C-labeled hyaluronic acid (HA) was subjected to acid hydrolysis with 1 M HCl—a harsh condition generating not only short, saturated oligomers (e.g., dimers and tetramers), but also promoting deacetylation. As shown in Figure S1 of Supporting Information, the deacetylated dimer exhibits a dominant peak at *m*/*z* 366.1447, corresponding to molecules fully labeled with ^13^C, and a secondary peak at *m*/*z* 365.1416, attributed to species containing a single ^12^C atom. This minor isotopologue arises from an isotopic impurity in the commercial ^13^C-glucose used for biosynthesis, which typically contains up to 1% unlabeled glucose. In contrast, the acetylated dimer displays three peaks (Figure 2). In addition to the ions at *m*/*z* 410.1614 and 409.1594, which correspond to fully ^13^C-labeled molecules and to those with a single ^12^C atom, respectively, a third peak is observed at *m*/*z* 408.1556. This peak is indicative of molecules containing two ^12^C atoms, consistent with partial labeling of the acetyl group and incomplete ^13^C incorporation during biosynthesis. MS/MS fragmentation provides further confirmation of this interpretation. For the precursor ion at *m*/*z* 409.1594, containing one ^12^C atom, fragmentation yields two isotopologues of the GlcA fragment at *m*/*z* 199.055 and 198.0526, separated by ∼1 Da.

**Figure 4:**
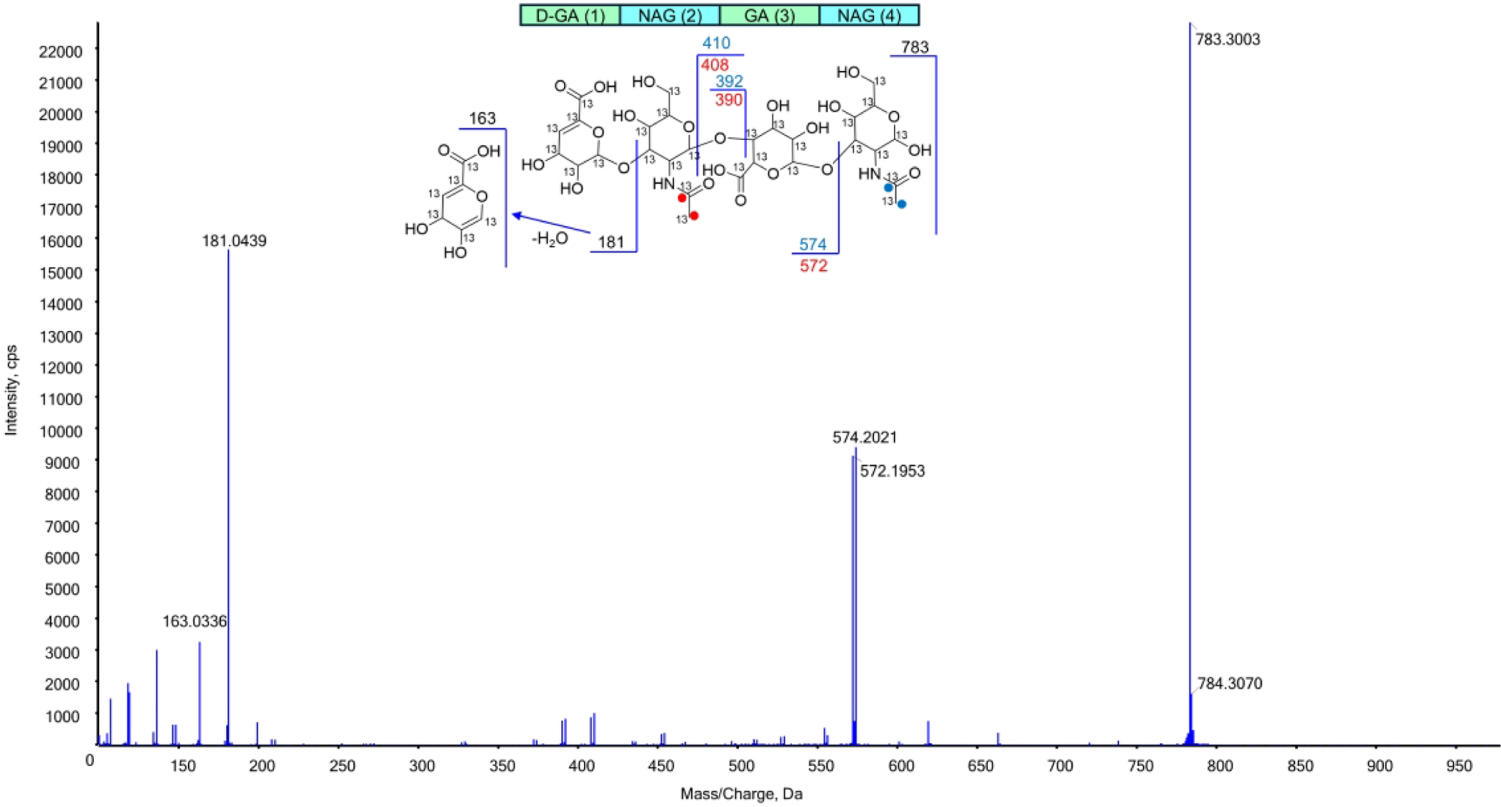
MS/MS spectrum of the ion at *m*/*z* = 783. Fragment attributions are displayed in the inset. The red/blue dots indicate possible ^13^C labeling, and the relative generated fragments are of the same color.

The relative intensities of these peaks reflect the probability of the ^12^C atom residing in the 6-carbon GlcA fragment (versus the 8-carbon GlcNAc moiety lost as a neutral). Based on this distribution, the fragment containing one ^12^C atom appears at lower intensity (∼40%) compared to the all-^13^C isotopologue (∼60%). Conversely, MS/MS analysis of the fully labeled precursor ion (*m*/*z* 410.1614) produces only a single GlcA fragment peak, as expected in the absence of ^12^C. Similarly, a single GlcA fragment is observed for the ion at *m*/*z* 408.1556, which lacks the acetyl group and is fully ^13^C-labeled. A 4-mer isotopic analogue containing 14 ^13^C-labeled carbon atoms out of a total of 28 was employed as a second internal standard (IS2) to normalize the mass spectrometry (MS) response. IS2 was generated by hydrolyzing 50%-^13^C-labeled hyaluronic acid (HA) using bovine testicular hyaluronidase (BTH). The ^13^C-labeled HA was produced by supplementing the fermentation medium with a 1:1 mixture of ^13^C-glucose and ^12^C-glucose. This approach yielded a heterogeneous population of HA molecules with varying degrees of ^13^C incorporation, as confirmed by the isotopic distribution of the Δ4-mer fragments obtained via enzymatic cleavage with RSK. As shown in Figure 2, panel C, the resulting Δ4-mer exhibited a complex isotopic pattern with species containing between 0 and 20 ^13^C atoms. Among these, the species containing 6, 8, 12, and 14 labeled carbon atoms were the most abundant. The Δ4-mer carrying 14 ^13^C atoms was initially selected as the IS2 (50%-13C-Δ4-mer). However, in addition to this dominant isotopologue, Δ4-mer species containing 0 and 28 ^13^C atoms were also released during hydrolysis of 50%-^13^C-HA. To prevent overlap between the isotopologues derived from IS2 and those originating from the sample HA and the first internal standard (IS1), BTH was specifically chosen due to its distinct hydrolytic mechanism compared to that of RSK. BTH cleaves HA to produce saturated oligosaccharides, which are shifted by +18 Da relative to the unsaturated fragments produced by RSK.^16^ This mass difference enables an unambiguous distinction between BTH-derived fragments and those generated by RSK, thereby eliminating any potential overlap with both the monoisotopic unlabeled Δ4-mer and the fully labeled (100% ^13^C) standard. Figure 5 illustrates the characteristic isotopic pattern of 4-mer isotopes obtained by the hydrolytic cleavage of 50%-^13^C-HA using BTH, reflecting the variable incorporation of ^13^C atoms. Notably, the ion at *m*/*z* 789.2748 contains 14 ^13^C atoms and corresponds to the 50%-^13^C-4-mer (IS2). This ion serves as the reference for normalizing the detector response.

**Figure 5:**
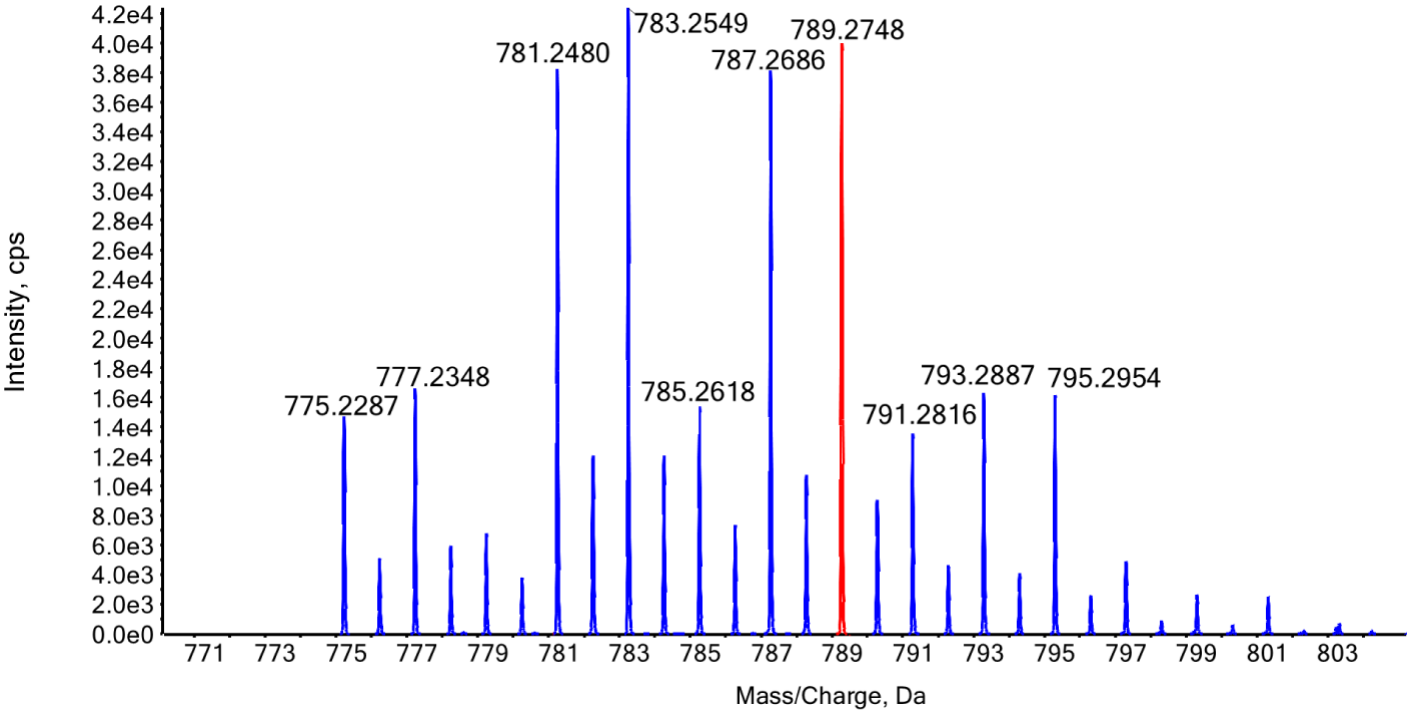
Isotopically labeled 4-mer resulting from enzymatic hydrolysis of HA, prepared by using a mixture of 50% ^12^C and ^13^C glucose, catalyzed by bovine hyaluronidase (BTH). The ion selected for the quantitative analysis is highlighted in red.

### HA Quantification in Biological Matrices

HA was quantified in two biological matrices with a high endogenous content of HA, namely bovine vitreous humor (BVH) and human synovial fluid (HSF). Since HA is an intrinsic component of both matrices and a true blank matrix is unavailable, the standard addition method was employed for quantitative analysis to account for and control the matrix effect. Depolymerization of HA is driven by the recombinant enzyme, which predominantly yields Δ4-mer as the main hydrolytic product. Figure S3 (Supporting Information) shows the relative abundances of monitored oligomers, ranging from dimers to 20-mers, formed by HA upon incubation with RSK, as determined by LC–ESI-MS. The Δ4-mer at *m*/*z* 757.2151 is the principal product, peaking after 24 h of incubation—a duration that was subsequently standardized for the sample preparation procedure. The amounts of HA added and the dilution factors applied to the original matrices were optimized to ensure good linearity without causing saturation of the MS signal. Internal standard (IS) concentrations were selected within a similar range to that of the analyte. Figure 6 presents representative MRM chromatograms of the Δ4-mer, Δ4-mer 100% ^13^C, and 4-mer 50% ^13^C following the addition of 0, 4, 12, and 20 μg/mL HA to BVH.

**Figure 6:**
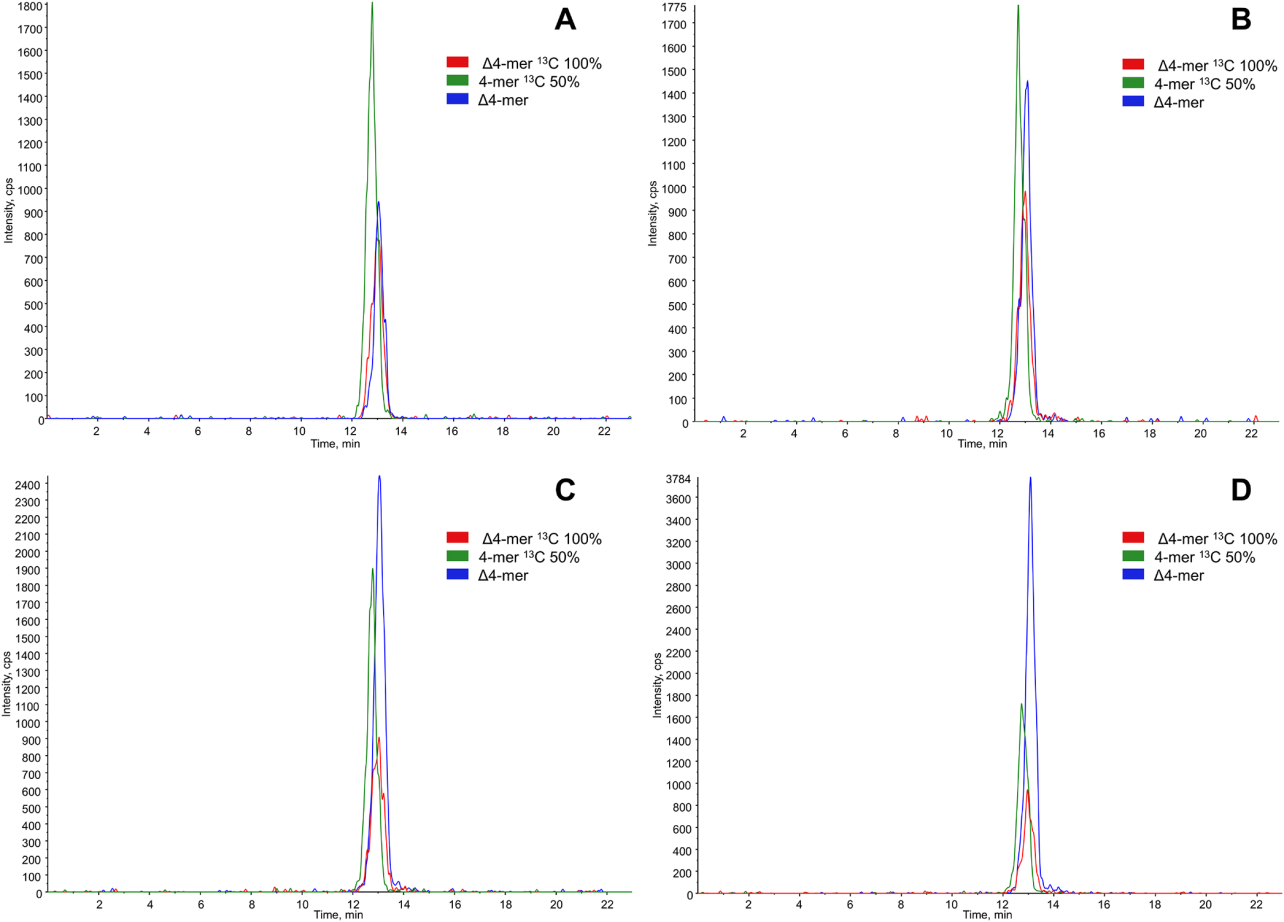
MRM chromatograms of Δ4-mer (blue), Δ4-mer 100% ^13^C (red), and 4-mer 50% ^13^C (green) with the addition of A) 0, B) 4, C) 12, and D) 20 μg/mL of HA to BVH.

Method validation was based on procedures reported in the literature,^14^ as no official guidelines are currently available for validating methods based on the standard addition approach. The parameters evaluated included: linearity of the calibration curves obtained through the standard addition method, estimated limits of detection (LOD) and quantification (LOQ), matrix effect, recovery, and both intraday and interday precision and accuracy. The calibration curve equations built by spiking the biological matrices with HA are reported in Table S1 of the Supporting Information, demonstrating good linearity, with *r* ^2^ values >0.99 for both BVH and HSF. Table 2 shows the mean estimated LOD and LOQ values that are comparable between the two matrices. Due to the endogenous nature of the analyte and the absence of a true blank matrix, it was not possible to prepare quality control samples spiked with low concentrations of the target compound, as typically required for conventional bioanalytical method validation. Therefore, the limits of detection (LOD) and quantification (LOQ) were estimated based on the signal-to-noise (S/N) ratio approach. It should be noted that in the present method, the tetramer oligomers are detected in their free form without the need for any derivatization process. However, if increased sensitivity is required, a derivatization step—as already proposed for HA oligomers—can be considered. Importantly, the inclusion of the two internal standards (IS) would also compensate for the variability introduced during the derivatization process.

**Table 2:** Mean ± SD of HA LOQ and LOD in BVH and HSF Expressed as μg/mL

Matrix	LOQ (μg/mL)	LOD (μg/mL)
BVH	0.491 ± 0.022	0.147 ± 0.007
HSF	0.475 ± 0.093	0.143 ± 0.028

Precision and accuracy were calculated at three concentration levels. In Table S2 and Table S3 of the Supporting Information are reported the values of accuracy (%) with the standard deviation and the relative standard deviation (RSD %) for the three concentrations tested for BVH and HSF, respectively, with and without the ISs. After one freeze–thaw cycle, the HA concentrations were 8.35% and 4.81% lower in BVH and HSF, respectively. After two cycles, the concentrations decreased by 14.77% and 13.56% for BVH and HSF, respectively, thus remaining within 20% variation with respect to the initial concentration for both the matrices. Table 3 reports the matrix effects and recovery values. With respect to recovery, values greater than 92% were observed for the analyte in both biological matrices. However, a significant matrix effect—approximately 50%—was detected in BVH. These findings highlight the critical importance of using the standard addition method for quantitative analysis when a matrix free of the analyte of interest is not available, as the presence of matrix effects can otherwise lead to inaccurate quantification.

**Table 3:** Recovery and Matrix Effect %

Matrix	Recovery (%)	Matrix effect (%)
BVH	99.13	53.23
HSF	92.98	113.15

To further evaluate the efficacy of the 100%-^13^C-labeled internal standard (IS1) in correcting variability arising from fluctuations in RSK enzymatic activity, a dedicated robustness experiment was performed by deliberately altering the enzyme concentration. Specifically, RSK was applied at three different activity levels: 1000 U/mL (50% reduction), 2000 U/mL (reference condition), and 5000 U/mL (2.5-fold increase). The resulting Δ4-mer peak areas were monitored to assess the effect of the enzyme concentration on hydrolysis efficiency. As expected, substantial variation in Δ4-mer production was observed across the three conditions, with a coefficient of variation (CV%) of 25.2%, highlighting the method’s sensitivity to enzyme fluctuations (Figure S4 of the Supporting Information). However, when IS1 was added and the analytical response was expressed as the ratio between the Δ4-mer and 100%-^13^C-Δ4-mer peak areas, the variations were normalized and CV% dropped markedly to 5.8%. This significant reduction demonstrates the normalization capability of IS1 in compensating for enzymatic variability. These results confirm the essential role of IS1 in the analytical protocol, not only to improve precision but also to enhance robustness under varying experimental conditions. Incorporating IS1 allows for more reliable and reproducible quantification of hyaluronic acid, especially in complex or variable biological matrices where enzymatic performance may be affected by sample-specific factors such as viscosity, protein content, or pH.

The concentrations (mean ± SD) of HA measured in BVH and HSF were 799.9 ± 144.4 and 1101.9 ± 151.4 μg/mL, respectively. According to data reported in the literature, the concentration of hyaluronic acid in bovine vitreous humor ranges from approximately 50 to 570 μg/mL. This wide variation is influenced by several factors, including the age of the animal and the specific physiological conditions of the tissue.^17^ In the analyzed sample, the amount was found to be significantly higher than values reported in the literature, but it should be noted that it is the matrix for which a high matrix effect was observed. Regarding human synovial fluid (HSF), HA concentrations are also known to vary significantly, typically ranging from 1 to 4 mg/mL in healthy individuals, and may decrease considerably in pathological conditions, such as osteoarthritis or rheumatoid arthritis. Therefore, the values obtained in this study for HSF are in line with those expected under normal physiological conditions.

## Conclusions

In this study, we developed and validated a bioanalytical method for the quantitative determination of hyaluronic acid (HA) in complex biological matrices, such as bovine vitreous humor and human synovial fluid. From an analytical chemistry perspective, the proposed strategy addresses several well-recognized limitations of the existing HA quantification approaches reported in the literature.

Current methods for HA analysis include immunochemical assays, chromatographic techniques, and mass spectrometry–based workflows. While ELISA-based methods allow direct measurement of polymeric HA, their quantitative reliability may be compromised by matrix effects, variable antibody affinity toward different molecular weight fractions, limited dynamic range, and challenges in standard harmonization.^18^ Conversely, chromatographic and MS-based approaches based on enzymatic depolymerization followed by disaccharide or oligosaccharide analysis have substantially improved selectivity, sensitivity, and throughput.^9,19^ However, these methods commonly rely on external calibration or quality control samples, with correction strategies addressing specific sources of variability—such as ionization efficiency or matrix effects^20^—while variability associated with extraction efficiency and enzymatic digestion is not directly normalized at the sample level.

The present method overcomes these analytical challenges by introducing isotopically labeled hyaluronic acid prior to extraction and enzymatic digestion, enabling direct normalization of sample preparation and hydrolysis efficiency. In addition, a second isotopically labeled internal standard at the instrumental level compensates for mass spectrometric response variability, while the standard addition approach effectively addresses pronounced matrix effects in the absence of analyte-free biological matrices.

Method validation demonstrated excellent linearity, low limits of detection, high recovery, and robust accuracy and precision across concentration levels. Overall, the proposed strategy enhances the quantitative robustness and traceability relative to existing methodologies by integrating isotopic normalization and matrix-adaptive calibration within a single analytical framework. This approach represents a reliable tool for absolute HA quantification in challenging biological matrices and supports its application in analytical, clinical, and translational research, including the investigation of pathological samples with altered HA metabolism, such as synovial fluids from osteoarthritic patients or tumor interstitial matrices.

## Supplementary Material



## Notes

Additional methods, MS spectra, chromatograms, and graphs (PDF)

## References

[1] Fraser J. R. E., Laurent T. C., Laurent U. B. G. (1997). Hyaluronan: Its Nature, Distribution,
Functions and Turnover. J. Intern. Med..

[2] Litwiniuk M., Krejner A., Speyrer M. S., Gauto A. R., Grzela T. (2016). Hyaluronic
Acid in Inflammation and Tissue Regeneration. Wounds.

[3] Tavianatou A. G., Caon I., Franchi M., Piperigkou Z., Galesso D., Karamanos N. K. (2019). Hyaluronan: Molecular Size-dependent
Signaling and Biological Functions in Inflammation and Cancer. FEBS J..

[4] Garantziotis S., Savani R. C. (2019). Hyaluronan Biology:
A Complex Balancing Act of Structure,
Function, Location and Context. Matrix Biol..

[5] Liu M., Tolg C., Turley E. (2019). Dissecting
the Dual Nature of Hyaluronan
in the Tumor Microenvironment. Front. Immunol..

[6] Monslow J., Govindaraju P., Puré E. (2015). Hyaluronan – a functional
and structural sweet spot in the tissue microenvironment. Front. Immunol..

[7] Šimek M., Lemr K., Hermannová M., Havlíček V. (2020). Analysis of
Hyaluronan and Its Derivatives Using Chromatographic and Mass Spectrometric
Techniques. Carbohydr. Polym..

[8] Rivas F., Erxleben D., Smith I., Rahbar E., DeAngelis P. L., Cowman M. K., Hall A. R. (2022). Methods
for Isolating and Analyzing
Physiological Hyaluronan: A Review. Am. J. Physiol.-Cell
Physiol..

[9] Volpi N., Galeotti F., Gatto F. (2025). High-Throughput Glycosaminoglycan
Extraction and UHPLC-MS/MS Quantification in Human Biofluids. Nat. Protoc..

[10] Chindaphan K., Wongravee K., Nhujak T., Dissayabutra T., Srisa-Art M. (2019). Online Preconcentration and Determination of Chondroitin
Sulfate, Dermatan Sulfate and Hyaluronic Acid in Biological and Cosmetic
Samples Using Capillary Electrophoresis. J.
Sep. Sci..

[11] Pittarello, M. ; Borile, F. ; Corsa, V. Process for the preparation and purification of the sodium salt of hyaluronic acid; WO 2,018,020,458 A1, 2018.

[12] Corsa, V. ; Negro, A. ; Vaccaro, S. ; Messina, L. Process for the production of hyaluronic acid in escherichia coli or bacillus subtilis; EP 2,614,088 A1, 2013.

[13] Cazzola, F. ; O’REGAN, M. ; Corsa, V. Culture medium and process for the preparation of high molecular weight hyaluronic acid; EP 0,716,688 B1, 2003.

[14] Hasegawa K., Minakata K., Suzuki M., Suzuki O. (2021). The Standard Addition
Method and Its Validation in Forensic Toxicology. Forensic Toxicol..

[15] Rampratap P., Lasorsa A., Perrone B., Van Der
Wel P. C. A., Walvoort M. T. C. (2023). Production of Isotopically Enriched
High Molecular Weight Hyaluronic Acid and Characterization by Solid-State
NMR. Carbohydr. Polym..

[16] Zhang Y.-S., Gong J.-S., Yao Z.-Y., Jiang J.-Y., Su C., Li H., Kang C.-L., Liu L., Xu Z.-H., Shi J.-S. (2022). Insights
into the Source, Mechanism and Biotechnological Applications of Hyaluronidases. Biotechnol. Adv..

[17] Shafaie S., Hutter V., Brown M. B., Cook M. T., Chau D. Y. S. (2018). Diffusion
through the Ex Vivo Vitreal Body – Bovine, Porcine, and Ovine
Models Are Poor Surrogates for the Human Vitreous. Int. J. Pharm..

[18] Wu Y., Zhao S., Wang J., Chen Y., Li H., Li J., Kan Y., Zhang T. (2024). Methods for Determining the Structure
and Physicochemical Properties of Hyaluronic Acid and Its Derivatives:
A Review. Int. J. Biol. Macromol..

[19] Hirai K., Ishii T., Aijima A., Yokota N., Miyamoto Y., Higashi K., Iwasaki Y., Ito R., Higashi N., Akiyama H. (2025). Development of a Method for Simultaneous
Analysis of
Glycosaminoglycan Disaccharides and Evaluating the Quality of Chondroitin
Sulfate and Hyaluronic Acid in Food Raw Materials. Food Chem.: x.

[20] Stancanelli E., Green D. E., Arnold K., Zhang J., Kong D., DeAngelis P. L., Liu J. (2024). Utility of Authentic ^13^C-Labeled Disaccharide to Calibrate Hyaluronan Content Measurements
by LC-MS. Proteoglycan Res..

