# Supplementary material for: Development of an Accurate Double Isotopic Standard LC–MS/MS Method for Hyaluronic Acid Quantification in Biological Matrices
Source: Anal Chem. 2026 Feb 11;98(7):5370–80. doi: 10.1021/acs.analchem.5c06285 (PMC12937047; doi:10.1021/acs.analchem.5c06285)
Supplement: Supplementary file 1 [file ac5c06285_si_001.pdf]

SUPPORTING INFORMATION

DEVELOPMENT OF AN ACCURATE DOUBLE ISOTOPIC STANDARD LC-MS/MS METHOD FOR HYALURONIC ACID QUANTIFICATION IN BIOLOGICAL MATRICES

Simone Manzi,[1] Alessandra Altomare,[1] Giacomo Mosconi,[1] Maria Serena Rossitto,[2] Luciano Messina,[2] Anna Gallo,[3] Marina Carini,[1] Giancarlo Aldini,[1] Giovanna Baron[1]*

[1] Department of Pharmaceutical Sciences (DISFARM), Università degli Studi di Milano, Via Mangiagalli 25, 20133 Milan, Italy

[2] Fidia Farmaceutici S.p.A., Contrada Pizzuta, 96017 Noto (SR), Italy.

[3] Fidia Farmaceutici S.p.A., via Ponte della Fabbrica 3/A, 35031 Abano Terme (PD), Italy.

TABLE OF CONTENTS

S1. Synthesis of HA isotopic standards.

S2. Enzymatic hydrolysis of HA by RSK

Table S1. Equations and correlation coefficients of the calibration curves.

Table S2. Accuracy (%) and RSD% intraday and interday in BVH with and without the use of the 2 ISs.

Table S3. Accuracy (%) and RSD% intraday and interday in HSF with and without the use of the 2 ISs.

Figure S1 - A) Full MS of the deacetylated dimer exhibits a dominant peak at *m/z* 366.1447, corresponding to molecules fully labeled with ^{13}C, and a secondary peak at *m/z* 365.1416, attributed to species containing a single ^{12}C atom. B) MS/MS spectrum of the deacetylated dimer at *m/z* 366.

Figure S2 - A) Full MS of the acetylated dimer exhibits a dominant peak at *m/z* 410.1614, corresponding to molecules fully labeled with ^{13}C, a secondary peak at *m/z* 409.1594, attributed to species containing a single ^{12}C atom, and a third peak at *m/z* 408.1556. B) MS/MS spectrum of the acetylated dimer ion at m/z 410.

Figure S3 - Relative abundances of oligomers obtained after RSK hydrolysis: in blue the 2-mer at 1.1 min (*m/z* 378.10363 as [M-H]-), in red the 4-mer at 2.4 min (*m/z* 757.21508 as $[M-H]^-$ and *m/z* 378.10363 as $[M-2H]^{2-}$, in blue), and in green the 6-mer at 5.9 min (*m/z* 567.65935 as $[M-2H]^{2-}$).

Figure S4 - Impact of IS1 addition on the robustness of the method: in blue are reported the areas of Δ4-mers produced by the different RSK concentration (1000, 2000 and 5000 U/mL). It is evident how the area is influenced by the RSK concentration. In red are reported the area ratios of Δ4-mers/100%-^{13}C-Δ4-mer, highlighting the importance of the IS1 to normalize the hydrolytic process.

S1. Synthesis of HA isotopic standards.

Labeled HAs were biosynthetically prepared using *Streptococcus equi* subsp. *equi* with *D*-glucose (U-$^{13}C_6$, 99%) as the carbon source. The strain used in this study is the property of Fidia Farmaceutici S.p.A. (Noto Unit collection) and belongs to the genus *Streptococcus* routinely used for HA production, species *equi* subsp. *equi*. Before the inoculum preparation for laboratory-scale fermentation, the strain was previously subjected to sensitization by growing on vegetable peptone agar (31g/L, VPA; Thermo Fisher Scientific, Italy) with 1% *D*-glucose (U-$^{13}C_6$, 99%, Spectra 2000 srl, Italy) under aerobic conditions at 37 °C for 16-18 h. To prepare the inoculum, 1 to 3 isolated colonies grown on VPA were used to inoculate 100 mL of vegetable peptone (31g/L, VP; Fisher Scientific, Italy). Incubation was performed with shaking at 200 rpm, as described above. This overnight culture was then used to inoculate 1L of productive medium consisting of Hy Soy Kerry (2 g/L, Fisher Scientific, Italy); SoyPeptone (2 g/L, HiMedia La-boratories GmbH, Germany); Na_2HPO_4, (6 g/L); KH2PO4, (3 g/L); NaCl, (0.5 g/L); NH4Cl, (1 g/L); $MgSO_4{\cdot}7H_2O$, (1mM); CaCl2 (1µM); histidine (0,0015%); leucine (0.004%); U-13C6 (50 g/L for 100% ^{13}C-labeled HA or 20 g/L and *D*-glucose 30g/L for 50% ^{13}C-labeled HA) and phenol red (5 mg/L), at pH 7.0. The incubation was extended for 24 h. Sterile water solution of 5 M NaOH was gradually added to maintain the pH at 7.0. After 24 h the fermentation broth was brought to pH = 4.5 by adding 1 M HCl solution with stirring and incubated at 90 °C for 3h, to depolymerize HA and inactivate the biomass, which was removed by centrifugation (8000 rpm, 30 min, 4 °C). The supernatant, which contained isotopic HA, was sterilized with 0.2 µm membrane filter and NaOH solution (20%) was added with shaking (200 rpm) to achieve a pH of 6.9-9-7.4. To a first HA purification from larger molecules like proteins, pigments, and other cellular debris, the Celite (10 g, diatomaceous fossil flour, Fisher Scientific, Italy) was added with shaking for 5 min. Moreover, the quaternary cetylpyridinium salt (7.5 g, CTP, Merck KGaA, Germany) was added with shaking and maintained for 10 min until the HA-CTP complex was formed. The HA-CTP was filtered (Gooch n.2) and the celite was washed 4-times with water (4 L). The supernatant, a solution containing HA isotopic was incubated for 16-18 °C at 30 °C in saline (0.3 NaCl, Merck KGaA, Germany) to obtain its sodium salt. After filtration (Gooch n.2 or 3), the supernatant was mixed with EDTA (23 mg, ethylenediaminetetraacetic acid, Merck KGaA, Germany) for cation complexation, and Diaion HP20LX resin (10 g, Fisher Scientific, Italy) for a further purification and kept at 30 °C for 16-18h with stirring. To reach the HA molecular weight of approximately 200 kDa the supernatant was recovered, treated with a NaOH solution (0.5 M) to a final concentration of 0.3 M, and kept at 40 °C for 4 h with stirring. The reaction was stopped by adding a 18% HCl solution to a pH of 8.8- 8.9 and the supernatant was collected by filtration (0.2 µm nylon filter, Sartorius Italy srl). The isotopic HA was precipitated by adding 2 volumes of EtOH and recovered by filtration. Subsequently, the polymer was dried in vacuum at 40°C for 48-72h. The powder was analyzed by gel permeation chromatography/size exclusion chromatography (GPC/SEC) using Viscotek TDA 302 systems, equipped with a triple detector: refractive index, low-angle laser light scattering, differential capillary viscometer.

S2. Enzymatic hydrolysis of HA by RSK

An aliquot of 100 µL was taken from a stock solution of 100%-13C labeled HA (3.4 mg/mL in water) or 50%-13C la-beled HA, to which 100 µL of acetate buffer (200 mM, 300 mM NaCl, pH 5.2) and 1.6 µL enzyme stock solution (RSK 100 U/mg, 100.696 U/mL) were added. After 24 hours incu-bation, 10 volumes of cold methanol were added and the sample kept for 30 minutes at - 20 °C, then centrifuged at 4 °C for 15 minutes at 15200 rpm, and the supernatant dried in under vacuum. The dry sample was taken up in 70 µL of 100 mM ammonium formate pH 3/ACN 50:50 (% v/v), vortexed, centrifuged for 15 minutes at room temperature, and the su-pernatant transferred in vial for the LC-ESI-MS analysis. 50%-13C labeled HA was also hydrolyzed by using BTH as follows: an aliquot of 500 µL was taken from a stock solution of 50% 13C labeled (10 mg/mL), to which 500 µL of acetate buffer (200 mM, 300 mM NaCl, pH 5.2) and 25 µL of BTH (25000 U/mL) were added to perform the hydrolysis at 37°C, 500 rpm, and incubated overnight. The hydrolysis was stopped by heating to 99° C for 20 minutes, then centrifuged for 30 minutes at 4°C, 15200 rpm. An aliquot of 20 µL was then diluted 1:4 in 100 mM ammonium formate pH 3/ACN 50:50 (% v/v) and analyzed by LC-HRMS. The remaining volume was then aliquoted and stored at -20 °C.

TABLES

Table S1. Equations and correlation coefficients of the calibration curves.

Matrix	Equation	Correlation coefficient (r^2)
**BVH**	**y = 2.975E-06x + 1.985E-05**	**0.9948**
**HSF**	**y = 3.362E-06x + 2.416E-05**	**0.9979**

Table S2. Accuracy (%) and RSD% intraday and interday in BVH with and without the use of the 2 ISs.

	**With IS1 and IS2**				**Without IS1 and IS2**			
	**Intraday (n=5)**		**Interday (n=5)**		**Intraday (n=5)**		**Interday (n=5)**	
**HA addition (µg/mL)**	%	RSD%	%	RSD%	%	RSD%	%	RSD%
**4**	89.13±2.72	3.05	93.26±18.42	19.78	111.49±10.59	9.5	114.17±9.50	8.32
**12**	93.34±5.14	5.5	91.17±6.04	6.62	107.51±1.78	1.66	116.61±12.63	10.83
**20**	101.40±5.72	5.64	96.16±11.49	11.95	100.57±4.19	4.17	105.24±6.11	5.81

Table S3. Accuracy (%) and RSD% intraday and interday in HSF with and without the use of the 2 ISs.

	**With IS1 and IS2**				**Without IS1 and IS2**			
	**Intraday (n=5)**		**Interday (n=5)**		**Intraday (n=5)**		**Interday (n=5)**	
**HA addition (ug/mL)**	%	RSD%	%	RSD%	%	RSD%	%	RSD%
**8**	102.02±9.10	8.92	95.34±12.51	13.13	85.84±2.61	3.04	88.98±4.71	5.3
**24**	104.87±1.39	1.33	102.89±6.53	6.35	94.95±2.49	2.63	95.80±2.59	2.7
**40**	97.70±2.52	2.58	98.30±10.30	10.48	99.54±5.48	5.51	89.64±6.78	7.56

FIGURES

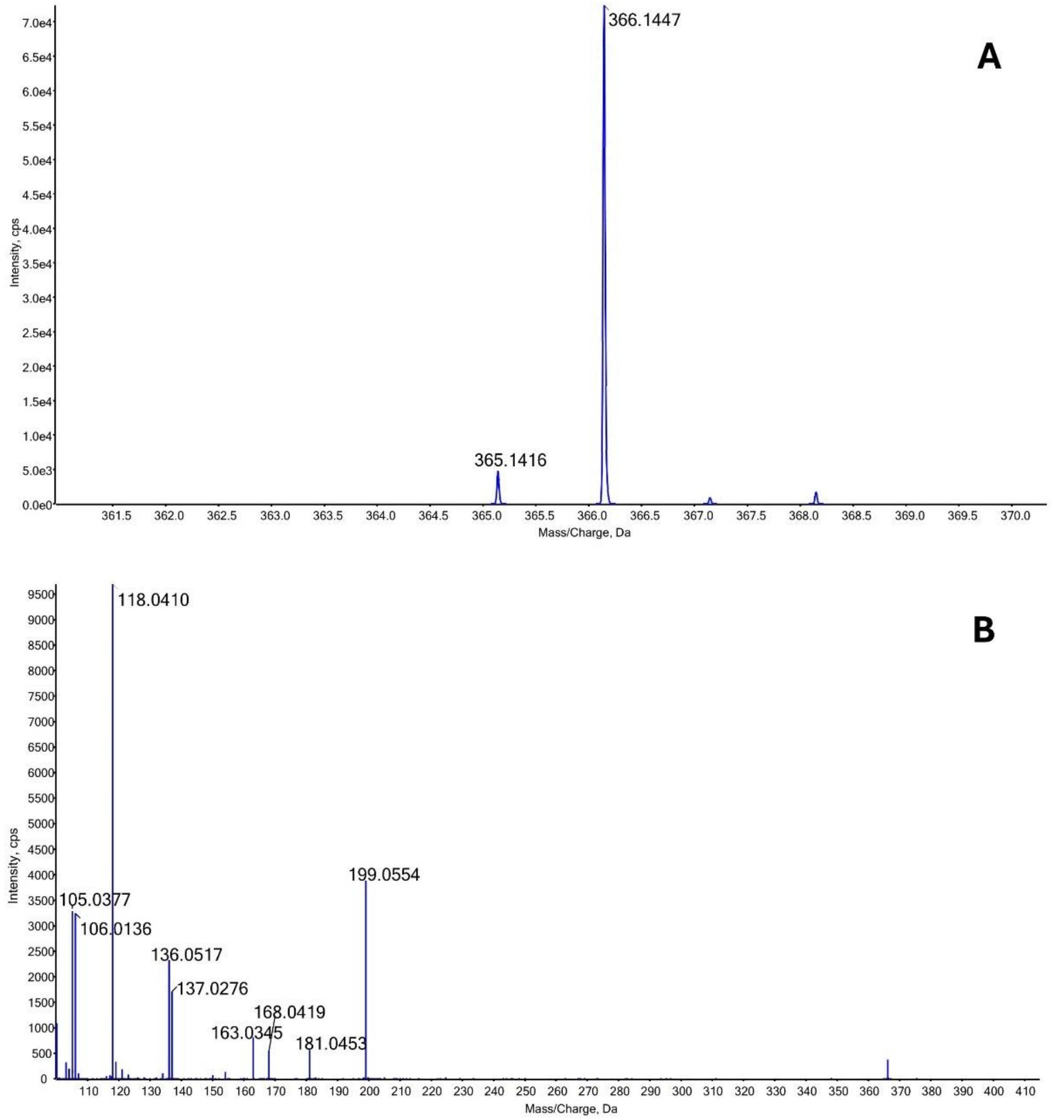

Figure S1 - A) Full MS of the deacetylated dimer exhibits a dominant peak at *m/z* 366.1447, corresponding to molecules fully labeled with ^{13}C, and a secondary peak at *m/z* 365.1416, attributed to species containing a single ^{12}C atom. B) MS/MS spectrum of the deacetylated dimer at *m/z* 366.

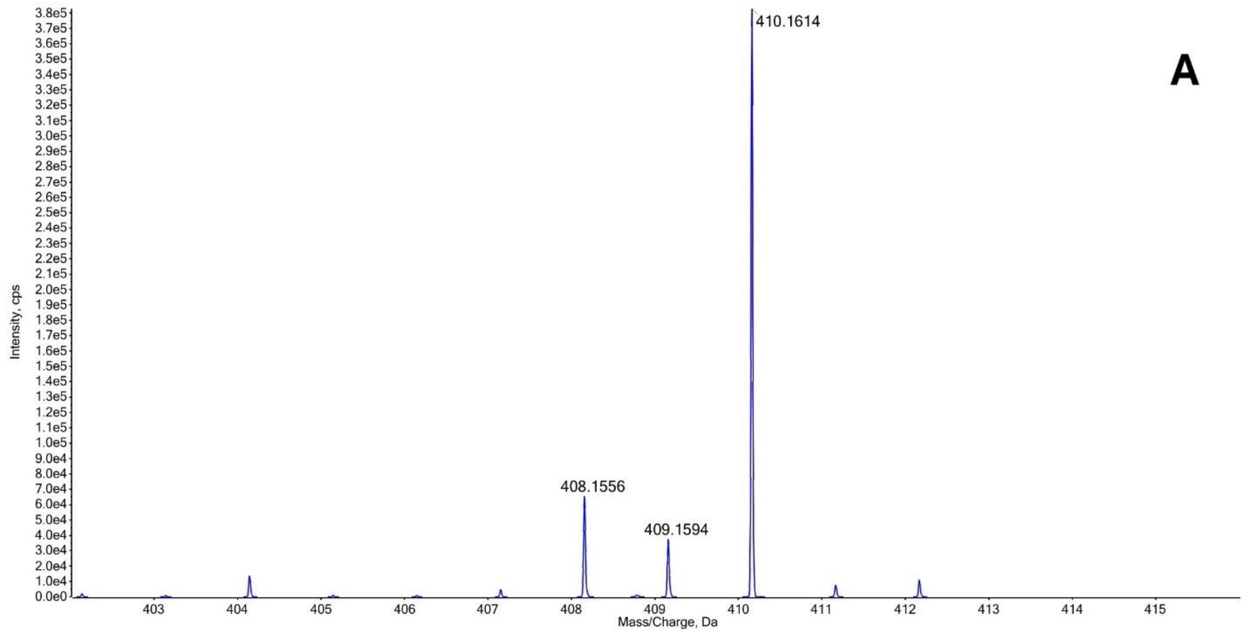

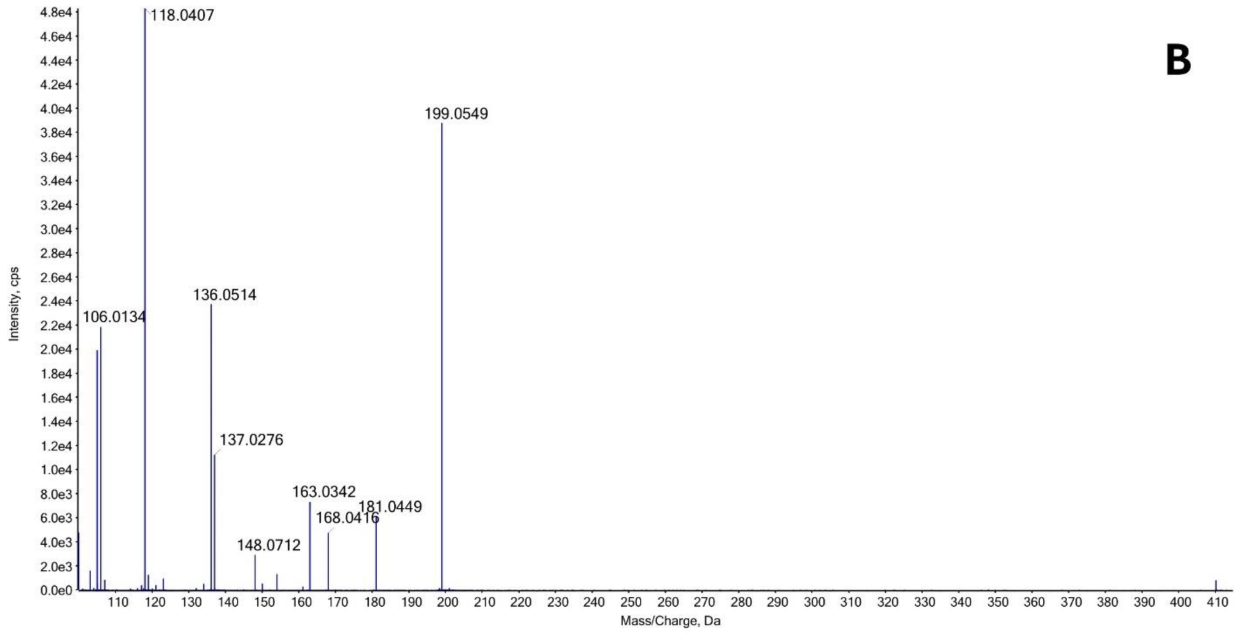

Figure S2 - A) Full MS of the acetylated dimer exhibits a dominant peak at *m/z* 410.1614, corresponding to molecules fully labeled with ^{13}C, a secondary peak at *m/z* 409.1594, attributed to species containing a single ^{12}C atom, and a third peak at *m/z* 408.1556. B) MS/MS spectrum of the acetylated dimer ion at *m/z* 410.

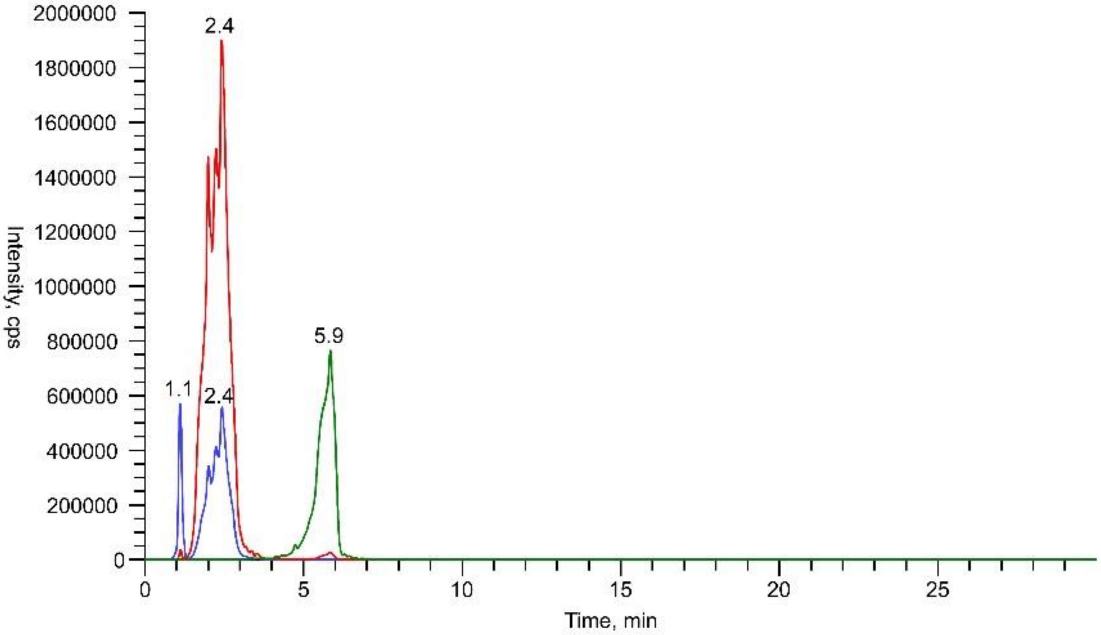

Figure S3 - Relative abundances of oligomers obtained after RSK hydrolysis: in blue the 2-mer at 1.1 min (m/z 378.10363 as $[M-H]^-$), in red the 4-mer at 2.4 min (*m/z* 757.21508 as $[M-H]^-$ and *m/z* 378.10363 as $[M-2H]^{2-}$, in blue), and in green the 6-mer at 5.9 min (*m/z* 567.65935 as $[M-2H]^{2-}$).

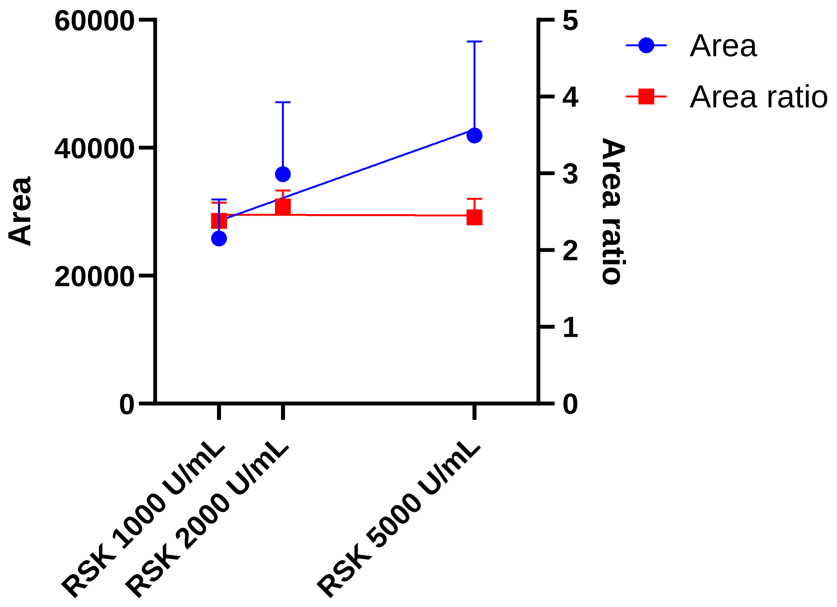

Figure S4 - Impact of IS1 addition on method robustness : blue symbols represent the peak areas of Δ4-mers produced at different RSK concentrations (1000, 2000 and 5000 U/mL). A clear dependence of the Δ4-mer peaks area on the RSK concentration is observed. Red symbols show the Δ4-mers/100%-^{13}C-Δ4-mer peak area ratios, highlighting the importance of the IS1 in normalizing the variability the hydrolytic process.
